# Parasitic Helminth-Derived microRNAs and Extracellular Vesicle Cargos as Biomarkers for Helminthic Infections

**DOI:** 10.3389/fcimb.2021.708952

**Published:** 2021-06-25

**Authors:** Yi Mu, Donald P. McManus, Catherine A. Gordon, Pengfei Cai

**Affiliations:** Molecular Parasitology Laboratory, Infectious Diseases Program, QIMR Berghofer Medical Research Institute, Brisbane, QLD, Australia

**Keywords:** helminth, microRNA, extracellular vesicles, biomarker, diagnosis

## Abstract

As an adaption to their complex lifecycles, helminth parasites garner a unique repertoire of genes at different developmental stages with subtle regulatory mechanisms. These parasitic worms release differential components such as microRNAs (miRNAs) and extracellular vesicles (EVs) as mediators which participate in the host-parasite interaction, immune regulation/evasion, and in governing processes associated with host infection. MiRNAs are small (~ 22-nucleotides) non-coding RNAs that regulate gene expression at the post-transcriptional level, and can exist in stable form in bodily fluids such as serum/plasma, urine, saliva and bile. In addition to reports focusing on the identification of miRNAs or in the probing of differentially expressed miRNA profiles in different development stages/sexes or in specific tissues, a number of studies have focused on the detection of helminth-derived miRNAs in the mammalian host circulatory system as diagnostic biomarkers. Extracellular vesicles (EVs), small membrane-surrounded structures secreted by a wide variety of cell types, contain rich cargos that are important in cell-cell communication. EVs have attracted wide attention due to their unique functional relevance in host-parasite interactions and for their potential value in translational applications such as biomarker discovery. In the current review, we discuss the status and potential of helminth parasite-derived circulating miRNAs and EV cargos as novel diagnostic tools.

## Introduction

Parasitic helminths, comprising the Phylum Platyhelminthes and the Phylum Nematoda, are regarded as some of the most prevalent human infectious agents in developing countries (Hotez et al., 2008). The most common human helminthiases are caused by soil-transmitted helminths, schistosomes (causative agents of schistosomiasis) and filarial worms, which cause onchocerciasis and lymphatic filariasis (Hotez et al., 2008). Approximately 8 million DALYs are lost annually due to these infections (Molyneux et al., 2017). As a result of the high prevalence and significant morbidity they cause in both humans and livestock animals, parasitic helminths represent an important global health problem and the cause of significant economic burden (Charlier et al., 2014). Developing effective strategies such as vaccines and accurate diagnostic tools for the prevention and control of helminth infections will be paramount in reducing the overall disease burden due to these parasites (Lustigman et al., 2012).

Small non-coding RNAs (sncRNAs) are a class of non-coding RNAs (ncRNA), which have been identified in a wide range of organisms including helminths (Cai et al., 2016). sncRNAs include housekeeping ncRNAs, such as small nuclear RNAs (snRNAs), transfer RNAs (tRNAs), and tRNAs-derived small RNAs (tsRNAs); and regulatory ncRNAs, such as microRNAs (miRNAs), endogenous short interfering RNAs (siRNAs), and PIWI-interacting RNAs (piRNAs) (Zhang et al., 2019). Due to advances in helminth genomics (Ghedin et al., 2007; The Schistosoma japonicum Genome Sequencing and Functional Analysis Consortium, 2009; Zheng et al., 2013; International Helminth Genomes Consortium, 2019) and high-throughput deep sequencing, a considerable number of miRNAs have been identified in a range of parasitic helminths (Hao et al., 2010; Poole et al., 2010; Cucher et al., 2015). Furthermore, miRNA expression profiles of different developmental stages and/or in specific tissues or cell types have been determined in some key helminth taxa (Cai et al., 2011; Cucher et al., 2011; Cai et al., 2013; Bai et al., 2014). These studies provide a solid basis for further functional and translational investigations of helminth miRNAs. Accumulating evidence also reveals that sncRNAs present in the circulation may play an important role in disease diagnosis and prognosis (Drury et al., 2017), particularly for cancer (Lan et al., 2015). The presence of helminth-derived miRNAs in the serum/plasma of helminth-infected definitive hosts has stimulated considerable interest in evaluating the potential of utilizing worm-derived miRNAs as diagnostic biomarkers for helminthiases (Cai et al., 2016; Ghalehnoei et al., 2020; Cucher et al., 2021).

Extracellular vesicles (EVs) are small membrane-bounded secreted vesicles that were previously considered a general mechanism for waste disposal by living systems including helminths, but are now recognized to be important cell-cell communicators, as they are able to transmit a wealth of bioactive cargos, such as proteins, lipids, glycans, DNA, messenger RNAs (mRNAs), small RNAs, and DNAs between cells (Yáñez-Mó et al., 2015). EVs are found in all bodily fluids including blood, urine, saliva, cerebrospinal fluid, milk and pleural effusion (Karin-Kujundzic et al., 2019) and they possess several key properties which underpin their potential as biomarkers, including 1) structural stability, 2) high abundance in plasma, and 3) the ability to alter their concentration and constitution under diverse conditions (Wu et al., 2019).

**Figure 1:**
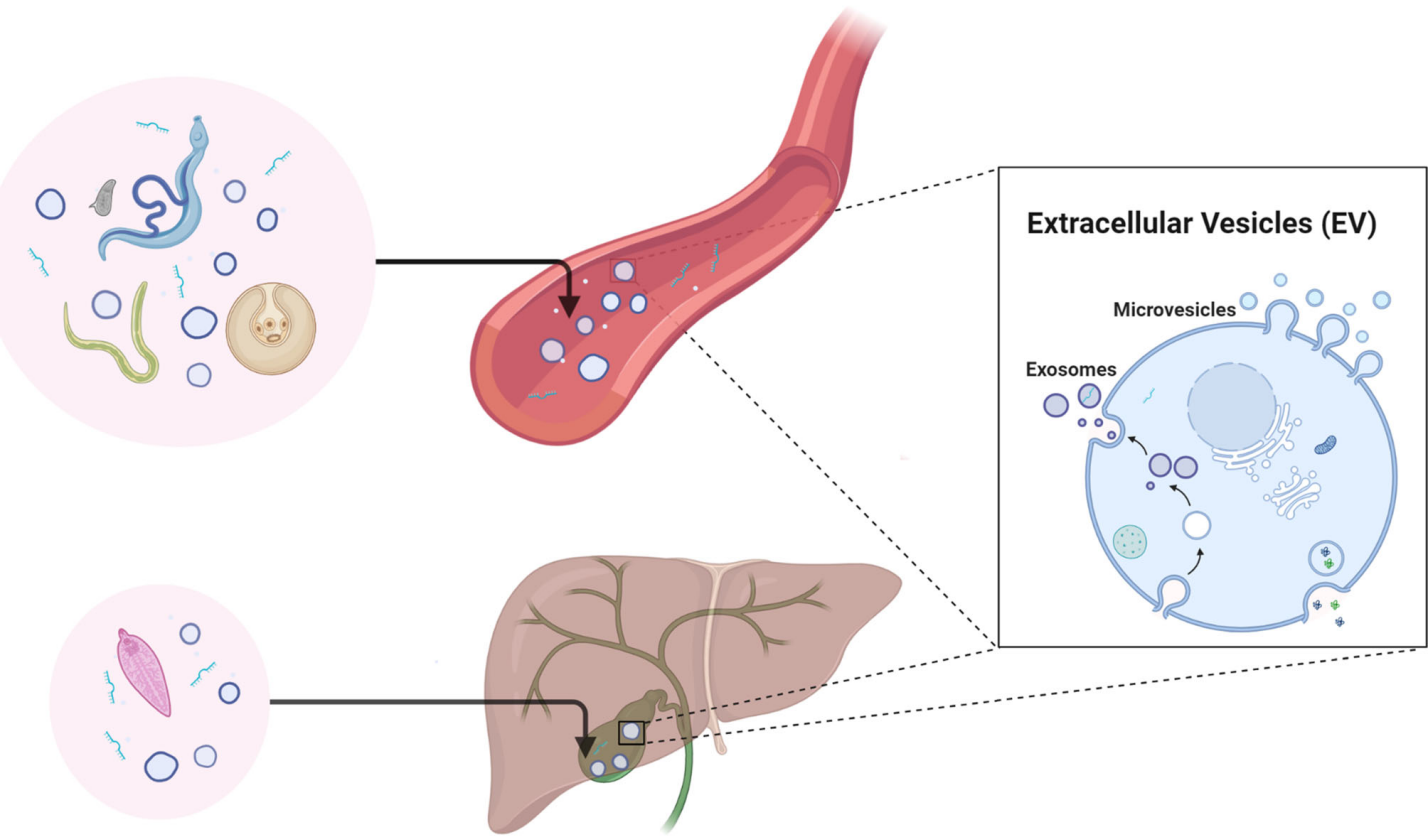
Diagrammatic representation of the secretion of helminth-derived non-vesicular extracellular miRNAs and/or EVs in the circulatory system and bile of the mammalian host. **Figure 1** was created with BioRender.com.

The application of specific EV molecules as biomarkers for the diagnosis and prognosis of diseases, including but not limited to cancer, have been widely reported and reviewed (Lai et al., 2017; Kosaka et al., 2019; Logozzi et al., 2020; Pang et al., 2020). Studies on helminth-derived EVs have been carried out in the last decade, ranging from the determination of their molecular composition (Sotillo et al., 2016; Samoil et al., 2018; Sotillo et al., 2020) to dissecting their regulatory roles in the host immune system/immunopathology (Buck et al., 2014; Eichenberger et al., 2018b; Wang et al., 2020a). However, there is still much to be explored regarding the applications of helminth-derived EVs, such as their potential in diagnosis, as therapeutics (Siles-Lucas et al., 2015), and as vaccines (Mekonnen et al., 2018). Accumulating evidence shows that parasitic helminths, particularly those that are blood dwelling, can actively release EVs into the host circulatory system and other bodily fluids (**Figure 1**) (Meningher et al., 2017; Ricciardi et al., 2021). These advances have led to the speculation that components within helminth-derived EVs may represent a potential source of biomarkers for helminthic diseases (Zakeri et al., 2018).

## Helminth-Derived miRNAs and EV Constituents in the Circulatory System as Potential Biomarkers

### Trematodes

There is increased interest in determining the potential of trematode-derived miRNAs as biomarkers of infection and disease. A number of studies have targeted the human schistosome blood flukes, the causative agents of schistosomiasis that afflicts more than 250 million people worldwide (McManus et al., 2020). Cheng et al. (2013) were the first to report deep sequencing of small RNA populations in the plasma of *Schistosoma japonicum*-infected rabbits and identified five schistosome-specific miRNA signatures (sja-bantam, sja-miR-3479, sja-miR-10, sja-miR-3096 and sja-miR-8185). Four of these five miRNAs were also confirmed in the plasma of *S*. *japonicum*-infected mice by RT-PCR. This study, for the first time, indicated that helminth-derived miRNAs had potential as biomarkers of helminthiases. In a subsequent low dosage cercarial infection animal model, sja-miR-277 and sja-miR-3479-3p, but not sja-bantam, were reliably detected in the sera of *S. japonicum*-infected BALB/c and C57BL/6 mice by RT-PCR (Cai et al., 2015). In addition, the serum levels of sja-miR-277 and sja-miR-3479-3p showed a positive correlation with hepatic egg burdens as well as the severity of liver fibrosis (Cai et al., 2015).

In a subsequent study, six miRNA candidates were validated with serum samples from a human cohort in a schistosomiasis-endemic area of the Philippines, which showed that two parasite-derived miRNAs (sja-miR-2b-5p and sja-miR-2c-5p) could be detected in infected individuals with a moderate diagnostic performance (sensitivity/specificity values of 66%/68% and 55%/80%, respectively) (Mu et al., 2020). Duplex and multiplex assays were also developed for the detection of schistosomal miRNAs for which the diagnostic performance was also moderate (Mu et al., 2020). These rather moderate diagnostic outcomes may have been the result of the low intensity infections in the study cohort as the endemic area experienced regular mass drug administration (MDA) for schistosomiasis. For the detection of another schistosome species, *S. mansoni*, three parasite-derived miRNAs (sma-miR-277, sma-miR-3479-3p and sma-bantam) were identified in the sera of *S. mansoni*-infected mice and patients from endemic areas in Zimbabwe and Uganda (Hoy et al., 2014). Detection of these miRNAs in human sera resulted in specificity/sensitivity values of 89%/80% and 80%/90%, respectively.

Elsewhere, miRNAs of *S. mansoni* and *S. haematobium* (sub-Saharan Africa) and *S. mekongi* (Laos) have been detected in the sera of returning travelers with schistosomiasis (diagnosis confirmed by egg detection or serology); the level of sma-bantam was able to distinguish infected individuals from healthy controls with a sensitivity of 66% and a specificity of 85%, respectively and with an area under the curve (AUC) of 0.786 (Meningher et al., 2017). Circulating miRNAs were also identified in buffaloes infected with *Fasciola gigantica*, a tropical liver fluke and the cause of fascioliasis; four worm-specific miRNAs, fgi-miR-87, fgi-miR-71, fgi-miR-124 and, the novel miR-1, were identified in the sera of infected animals by deep sequencing (Guo and Guo, 2019).

A number of EV components, including miRNAs and proteins, have been identified in different trematode species (Fromm et al., 2015; Sotillo et al., 2016; Zhu et al., 2016; Fromm et al., 2017; Samoil et al., 2018; Mekonnen et al., 2020; Ovchinnikov et al., 2020), and these can provide additional biomarkers for the detection of fluke infections. Meningher et al. (2017) explored whether miRNAs of helminth origin in serum EVs could be biomarker candidates for the diagnosis of schistosome infection in returning travelers. The authors confirmed that four schistosomal miRNAs (Bantam, miR-2c-3p, miR-3488 and miR-2a-5p) in serum EVs showed diagnostic potential, with the three former miRNAs exhibiting an AUC > 0.91. Two of the EV-derived miRNAs, bantam and miR-2c-3p showed a sensitivity/specificity of 85.71%/94.12% and 85%/93.75%, respectively. Notably, a proteomic analysis of adult *S. mansoni* worm EVs (Sotillo et al., 2016) showed that some antigens were present in the schistosome vesicles, including the vaccine candidate antigen Sm-TSP-2 and the ortholog (the saposin containing protein, Smp_130100) of a previously described *S. japonicum* diagnostic candidate, SjSPA4 (Cai et al., 2017). A study on the EVs released by *Fasciola hepatica*, the major cause of human fascioliasis and an emerging zoonotic pathogen, revealed the presence of the diagnostic antigen cathepsin L1 (Marcilla et al., 2012; Cwiklinski et al., 2015; Sarkari and Khabisi, 2017). It has been suggested that liver flukes can secret EVs into host bile (Marcilla et al., 2012; Chaiyadet et al., 2015), raising the possibility that the molecular information within EVs could be utilized for the diagnosis of fascioliasis.

### Cestodes

Tapeworm-derived miRNAs are stably detectable in the serum/plasma of mammalian hosts during infection even though these organisms do not reside in the blood vascular system. With *Echinococcus multilocularis*, which causes the very serious alveolar echinococcosis (AE) in humans, deep sequencing showed that seven parasite-specific miRNAs were detectable in the sera of mice infected with this tapeworm; two of the miRNAs, emu-miR-10 and emu-miR-227, were specifically amplified by RT-PCR, and thus may have potential as novel biomarkers for the diagnosis of AE (Guo and Zheng, 2017). In contrast, a more recent study exploring extracellular RNAs (ex-RNAs) produced by the metacestode stage of *E. multilocularis* found, using high throughput RNA-sequencing and RT-qPCR, that two miRNAs, miR-71-5p and miR-4989-3p, were secreted *in vitro* by the metacestode stage of *E*. *multilocularis*; however, although these two components were detectable in medium in which the parasites were cultured they were not found in the plasma/sera collected from a small number of patients with AE or cystic echinococcosis (CE) (due to E. granulosus) with hepatic location (Ancarola et al., 2020). Somewhat more encouraging, however, were results obtained by Alizadeh et al. (2020) who showed that the levels of two circulating worm-specific miRNAs (egr-miR-71 and egr-let-7) were detectable in the plasma of patients infected with *E. granulosus*, compared with uninfected individuals. Furthermore, the expression levels of both miRNAs declined substantially at three and six months post-cystectomy surgery to remove the echinococcal cysts, indicating that these miRNAs may represent promising novel biomarkers for the early diagnosis and monitoring of CE (Alizadeh et al., 2020).

The composition of EVs, such as small RNA and/or protein profiles is available for some cestode species (Wang et al., 2020b). In *E. granulosus*, studies were carried out on EVs isolated from hydatid cyst fluid (HCF) directly from hosts with CE (Siles-Lucas et al., 2017; Zhou et al., 2019) and cultured protoscoleces (Nicolao et al., 2019), leading to the identification of highly immunogenic antigens, such as antigen 5, Antigen B, P29 and endophilin-1. In *E. multilocularis*, Ding et al. (2019) identified 18 miRNAs from metacestode EVs, within which the top four expressed (emu-miR-71-5p, -let-7-5p, -miR-4989-5p and -miR-10-5p) and emu-miR-2c-3p also were detectable in the sera of parasite-infected mice. Notably, one of the threonine tRNA-derived small sequences positioned at the 5’ end has a dominant read count higher than that the sum of read counts of all 18 miRNAs, indicating that small RNAs, such as tsRNAs, may be better diagnostic biomarkers than miRNAs in EVs.

Another investigation confirmed EV production in different cestodes including *Taenia crassiceps*, *Mesocestoides corti* and *E. multilocularis* (Ancarola et al., 2017). However, unlike *T. crassiceps* and *M. corti* metacestodes, the *in vitro* culture of *E. multilocularis* metacestodes did not release EVs into the culture medium (Ancarola et al., 2017). This outcome conflicted with the observations of another group (Zheng et al., 2017) who were able to identify EVs in culture supernatants of *E. multilocularis* metacestodes. Ancarola et al. (2017) hypothesized that the laminated layer of larval cysts, which is a specialized extracellular matrix found only in members of the genus *Echinococcus*, acts as a barrier to EV release. However, there is still a possibility that metacestode EVs may be in contact with the host in the early stages of development, when the laminated layer is still not formed or is incipient, and/or when the laminated layer undergoes rupture due to metacestode ageing or chemotherapy treatment (Ancarola et al., 2017). Taken together, these studies provide a stepping-stone for the rational search of EV constituents for improved diagnosis of cestode infections.

### Nematodes

A number of studies on several species including *Loa loa*, *Dirofilaria immitis, Onchocerca ochengi* and *O. volvulus* demonstrate that parasitic nematode-derived miRNAs are actively released into the host circulatory system (Tritten et al., 2014a; Tritten et al., 2014b; Quintana et al., 2015). In a further study evaluating the circulating miRNAs released by *Angiostrongylus cantonensis* (the cause of eosinophilic meningoencephalitis) as potential biomarkers of infection, Chen et al. (2014) found that the level of aca-miR-146a in serum was significantly higher in *A. cantonensis*-infected mice compared with uninfected control animals, having an area under the curve value of 0.90 determined by receiver operating characteristic curve analysis. The diagnostic performance of aca-miR-146a was further assessed with human serum samples and showed a sensitivity of 83% and a specificity of 86.7%, respectively in 30 patients with proven angiostrongyliasis compared with 30 healthy individuals. Two independent studies confirmed the presence of a panel of *Onchocerca*-derived miRNA signatures in the nodular fluid and the plasma from *O. ochengi*-infected bovines (Tritten et al., 2014b; Quintana et al., 2015). Nematode-derived miRNAs, including miR-100a/d, lin-4 and miR-71, have also been detected in the serum/plasma of *O. volvulus*-infected humans (Quintana et al., 2015). However, further studies have demonstrated that the levels of *O. volvulus* miRNAs in human blood are too low to be employed as biomarkers for the detection of infection or treatment monitoring, even using locked nucleic acids (LNA)-based RT-qPCR analysis (Lagatie et al., 2017; Macfarlane et al., 2020).

Some studies have analyzed the composition of nematode-derived EVs (Buck et al., 2014) that constitute the basis for developing novel EV-targeted diagnostic tools for blood-dwelling and blood-feeding nematodes. A recent study exploring the regulatory roles of the EVs released from *Brugia malayi* microfilariae on the host innate immune system, demonstrated the presence of 576 proteins and a unique miRNA profile in the EVs (Ricciardi et al., 2021); these EV components should be pursued further as they may include markers with diagnostic potential. Hookworms are gut dwelling, blood-feeding nematodes affecting approximately 600 million people globally. Proteomics and RNAseq analysis of EVs from the rodent parasite *Nippostrongylus brasiliensis*, a model for human hookworm infection, identified 81 proteins, including 27 sperm-coating protein-like extracellular proteins in addition to those frequently found in exosomes (like tetraspanin, enolase, 14-3-3 protein, and heat shock proteins) and 52 miRNAs (Eichenberger et al., 2018a); these again warrant further study as potential diagnostic markers.

## Current Challenges and Future Perspectives

While there have been a number of recent studies utilizing circulating miRNAs for the diagnosis of helminthic infections, the results have been mixed and interpretation of results with miRNA raises some challenges/problems. 1) A low sample size was employed in most of the studies to date, which may lead to biased results. 2) Many promising reports involved data obtained in animal models, contrasting with the more challenging diagnostic application using human clinical samples, particularly those obtained in areas with low prevalence and infection intensity. When used for the detection of low intensity infections, the accuracy of detecting circulating miRNAs may be improved by using an optimized reverse transcription step, normalization methods (Deng et al., 2019; Cai et al., 2020) and/or more specific LNA primers. 3) The detection of helminth-derived miRNAs in circulation may be subject to the similar issues inherent to the detection of antigen and circulating cell-free DNA in the host that usually depends on an active infection. Indeed, the kinetics of decline in miRNA levels in the circulation after drug administration is still unclear, both in animal experiments and in humans after drug treatment. 4) No helminth-derived miRNA family member has emerged as a common biomarker for helminthic infections.

The recent identification of parasitic helminth-derived EV constituents (Sotillo et al., 2020) has provided new impetus for development novel EV-based diagnostic methods for helminthiasis, but the area is in its infancy due to the fact that researchers are presently confounded by the relative dearth of protocols for the isolation and enrichment of EVs released by parasitic helminths from those derived from their human and animal hosts. In this regard, a method has been developed for the rapid differentiation of host and parasite exosome vesicles using a microfluidic photonic crystal biosensor (Wang et al., 2018). Furthermore, it has been shown there is substantially increased enrichment of the ether lipid plasmalogen in parasite exosomes versus those derived from the mammalian host (Simbari et al., 2016), pointing the way for improved differentiation and purification of helminth parasite EVs. Different EV isolation methods may affect the outcome of the EV-based diagnostics; accordingly, strict methodological guidelines should be followed (Coumans et al., 2017; Thery et al., 2018). In addition, whereas the costs for developing diagnostic tools targeting circulating miRNA and EV components such as sncRNAs and proteins are high, expenditure could be reduced substantially if multiplex or high-through-put assays targeting multiple helminths and/or non-helminth pathogens are developed for simultaneous application (Sanprasert et al., 2019).

## Concluding Remarks

Parasitic worm-derived miRNAs/EVs play key roles in the development, host-parasite interplay and parasitic establishment of helminths, but their presence in the host circulatory system means they could also provide novel targets for parasite diagnosis. Current evidence confirms their utility in helminth diagnosis based on the detection of circulating helminth miRNAs for some taxa in the serum/plasma of mammalian hosts, but optimization steps are still needed to improve the performance of these assays. Diagnostic tools such as qPCR, serology, mass spectrometry, and next-generation sequencing targeting the contents (e.g. proteins and small RNAs including miRNAs and tsRNAs) encapsulated in worm EVs, can be applied for the diagnosis of parasitic worms, especially for blood-borne helminths such as *Schistosoma* spp., *B. malayi* (microfilarial stage), *L. loa*, *D. immitis* and *Onchocerca* spp., and blood-deeding helminths such as hookworms, once suitable enrichment of helminth-derived EVs can be achieved. Taking all the information currently available, it should be feasible to develop novel supplementary methods that target parasitic worm-derived miRNAs and EVs for the diagnosis of a number of important helminth parasites.

## Author Contributions

This manuscript was conceptualized by PC, drafted by YM, and revised by DM, CG, and PC. All authors contributed to the article and approved the submitted version.

## Funding

This work was funded by the National Health and Medical Research Council (NHMRC) of Australia (ID: APP1160046, APP1102926, APP1037304, APP1098244 and APP1194462). DPM is a NHMRC Leadership Fellow and Senior Scientist at QIMRB. The funders had no role in study design, data collection and analysis, decision to publish, or preparation of the manuscript.

## Conflict of Interest

The authors declare that the research was conducted in the absence of any commercial or financial relationships that could be construed as a potential conflict of interest.
